# Commentary: Long-term Practice with Domain-Specific Task Constraints Influences Perceptual Skills

**DOI:** 10.3389/fpsyg.2018.01214

**Published:** 2018-07-18

**Authors:** Christopher Yiannaki, Christopher Carling, Dave Collins

**Affiliations:** ^1^Centre for Research in Sports Performance, Myerscough College, Preston, United Kingdom; ^2^School of Sport and Wellbeing, Institute of Coaching and Performance, University of Central Lancashire, Preston, United Kingdom

**Keywords:** football, small-sided games, talent, skill acquisition, futsal, constraints

In our opinion, a recent article by Oppici et al. (2017) has generated interesting debate, enhancing the profile of futsal as a contemporary area for scientific research. Crucially for the purposes of this commentary, its findings can be discussed in relation to the acquisition and transfer of skills to 11 a-side soccer as part of talent development schemes (Travassos et al., 2017).

Research on Small Sided Games (SSG) has received wide coverage in the scientific literature (Aguiar et al., 2012; Halouani et al., 2014). The benefits of SSG in preparing players physically and technically for 11-aside soccer are widely recognized. However, specific constraints notably related to pitch size can directly impact on skill acquisition and subsequent applications to the 11-aside format (Davids et al., 2008; Vilar et al., 2014). In relation to the SSG “futsal,” there is a need for researchers to determine the impact of the specific constraints to which futsal exposes participants, thereby providing knowledge on its potential for transferal and acquisition of soccer-specific skills. Work by Oppici et al. (2017) has sought to analyse some of these components providing knowledge on the impact of futsal “*constraints*.” Their research adds empirical evidence to theoretical arguments on the potential for skills transfer recently presented (Travassos et al., 2017; Yiannaki et al., 2018).

The authors acknowledge the importance of considering the constraints of futsal and its potential for skill acquisition by focusing on perceptual skills and passing ability, with comparisons made concomitantly with soccer. The methodological approach employed sought to examine futsal in a positivist scientific paradigm, with control of experimental conditions. The authors utilized 6v6 tasks with pitch dimensions adjusted to create two “*domain specific tasks”* with “*Individual Playing Areas”* (IPA) for futsal (36 m^2^/player) and soccer (86 m^2^/player) accordingly. Broadly, results suggest that futsal players direct their gaze toward other players more during ball reception, whereas comparatively soccer players scan more “off the ball.” Authors speculate that observed behaviors are a direct consequence of sport specific skills acquisition. The benefit of this design is the factual objective nature of data, with variables closely controlled. However, we feel that additional research is needed before these findings can be used to inform the potential use of futsal as a skill development tool for 11-aside soccer. In our opinion, breaking down both sports into 6v6 versions and modifying pitch dimensions reduces the ecological validity of findings. In senior soccer, 11v11 is typically played and 5v5 in futsal. Through their epistemological approach, Oppici et al. (2017) sought to create controlled domain-specific practices, notably with respect to passing techniques. In order to justify pitch dimensions and IPA, the authors utilized a previous methodological approach (Fradua et al., 2013). However, the dynamic and changeable nature of the strategic and tactical demands in 11v11 soccer means that “IPAs” vary greatly. Thus, whilst their study created a mathematically controlled, domain-specific practice, the modified versions lack real-world reflection of match play. Inferences and potential applicability to the 11-aside game, therefore, carries limitations. An additional point of discussion is the mean age of participants used (13.6 years). The impact of pitch dimensions on determining physical and technical demands specifically in youth soccer has previously received attention (Castellano et al., 2016; Moreira et al., 2016). Numerous “National Governing Bodies” dictate the need for small, age-appropriate pitch sizes. In England, for example, “The Football Associaton” recommend reduced dimensions (U13/14 = 82.3 m × 50.3 m vs. Adult = 100.6 m × 64.0 m) (The FA, 2012), and smaller age appropriate soccer balls and goalposts. Further to these limitations, no offside rule was utilized in the soccer test. This again queries the potential generalisability of the observed behaviors and promotes the need for additional research that considers official rules, equipment and facilities, whilst is mindful of participant ages. Reflection on instrumentation is also necessary. In order to assess visual search strategies Oppici et al. (2017) utilized a sports camera method to track visual gaze behaviors, citing a paper by Pluijms et al. (2016) to justify this methodology. While this paper validates the approach for sailing, no evidence is available regarding its generalisability for invasion sports. Pluijms et al. (2016) also acknowledge that participants might feel “constrained” by this method thereby influencing behaviors observed.

In our opinion, the work by Oppici et al. (2017) provides useful preliminary information yet we feel that future studies should consider adopting a contrasting epistemological approach, examining behaviors in futsal and soccer in more authentic environments. According to Abbott and Collins (2004), in certain circumstances, quantitative data receives too greater importance as a consequence of the ability to measure it, as opposed to measuring behaviors which have clear causative relationships to actual game demands (Carling and Collins, 2014). Without representative design, behaviors that emerge may not be functional and realistic in dynamic sports performance environments (McGarry et al., 2013). Indeed, representative design (Phillips et al., 2010) would complement the initial research by Oppici et al., offering improved ecological validity and a more well-rounded understanding of futsal.

Consequentially, future research should examine authentic futsal match play. Performance analysis (PA) offers a methodological approach to provide objective statistics, with authors suggesting it provides a more naturalistic observation of authentic behaviors (McGarry et al., 2013). Existing futsal work (Milligan et al., 2002; Sturgess, 2017) could guide researchers in establishing observable, naturalistic behaviors in order to quantify commonalities in futsal and soccer performance. Reflecting on the work by Oppici et al., this research should take place inclusive of all official rules, pitch dimensions, and equipment, whilst being age appropriate for the participants and without hindrance from instrumentation. This analysis should take place during competitive match play at different levels, utilizing participants who are likely to display sports specific behaviors acquired through accumulating hours of domain specific practice, whilst improving the generalisability of conclusions (Swann et al., 2015). Research should also consider physical, physiological, technical and tactical parameters, allowing for a holistic examination of futsal. This data could help to determine the levels of similarity between futsal and soccer, which is crucial in facilitating skills transfer (Causer and Ford, 2014), and could provide both an applied and a theoretical basis to support the potential transfer of skills between the sports.

Future research should also consider the qualitative perceptions of relevant stakeholders in both game formats, allowing for a mixed methodological approach in answering some of the questions generated by by Oppici et al. Research such as that by Mills et al. (2012) suggests that analysis of the perspectives of coaches is under-researched, and often limited by sample size, and coach standard. This approach could provide a further opportunity for researchers, broadening the epistemological approaches, and creating richness in evidence whilst enhancing impact. Finally, future research should consider futsal intervention studies using randomized, controlled experimental designs. This will allow for the isolation of authentic futsal as a training aid for soccer, whilst measuring performance in skills/fitness testing (Russell and Kingsley, 2011) and PA of match play (McGarry et al., 2013).

## Author contributions

CY, CC and DC have contributed to all stages of the design and production of this general commentary.

### Conflict of interest statement

The authors declare that the research was conducted in the absence of any commercial or financial relationships that could be construed as a potential conflict of interest.
